# Molecular epidemiological investigations of plague in Eastern Province of Zambia

**DOI:** 10.1186/s12866-017-1146-8

**Published:** 2018-01-04

**Authors:** Stanley S. Nyirenda, Bernard M. Hang′ombe, Edgar Simulundu, Evans Mulenga, Ladslav Moonga, Robert S. Machang′u, Gerald Misinzo, Bukheti S. Kilonzo

**Affiliations:** 1 Central Veterinary Research Institute, P.O. BOX 33980, Balmoral, Lusaka Zambia; 2 Department of Microbiology, Parasitology and Biotechnology, Sokoine University of Agriculture, Morogoro, Tanzania; 3 Department of Paraclinical Studies, School of Veterinary Medicine, The University of Zambia, Lusaka, Zambia; 4 Department of Disease Control, School of Veterinary Medicine, The University of Zambia, Lusaka, Zambia; 5 Pest Management Centre, Sokoine University of Agriculture, Morogoro, Tanzania

**Keywords:** *Yersinia pestis*, Plague, Phylogenetic analysis, Zambia

## Abstract

**Background:**

Plague is a flea-borne zoonotic and invasive disease caused by a gram negative coccobacillus bacterium called *Yersinia pestis*. Plague has caused three devastating pandemics globally namely: the Justinian, Black Death and Oriental plague. The disease in the Eastern Province of Zambia has been reported in Nyimba and Sinda Districts in the past 15 years. The aim of this study was to investigate the molecular epidemiology of plague in the two affected districts. Polymerase Chain Reaction (PCR), targeting Plasminogen activator gene (*pla* gene) of *Y. pestis*, was performed on suspected human bubo aspirates (*n* = 7), rodents (*n* = 216), shrews (*n* = 27) and fleas (*n* = 1494). Of these, one positive sample from each source or host was subjected to sequencing followed by phylogenetic analysis.

**Results:**

The plasminogen activator gene (*pla* gene) of *Y. pestis* was detected in 42.8% bubo aspirates, 6.9% rodents, 3.7% shrew and 0.8% fleas. The fleas were from pigs (*n* = 4), goats (*n* = 5) and rodents (*n* = 3). The sequencing and phylogenetic analysis suggested that the *pla* gene of *Y. pestis* in Nyimba and Sinda was similar and the isolates demonstrated a high degree of evolutionary relationship with Antiqua strains from the Republic of Congo and Kenya.

**Conclusion:**

It can be concluded that *pla* gene of *Y. pestis* was present in various hosts in the two districts and the strains circulating in each district were similar and resembles those in the Republic of Congo and Kenya.

**Electronic supplementary material:**

The online version of this article (10.1186/s12866-017-1146-8) contains supplementary material, which is available to authorized users.

## Background

Plague is a flea-borne zoonotic disease caused by *Yersinia pestis*, a gram-negative coccobacillus, non-motile and non-spore-forming bacterium. The genus *Yersinia* is a member of the family *Enterobacteriaceae*, which consists of 11 species, of which *Y. pestis*, *Y. pseudotuberculosis* and *Y. enterocolitica* are human pathogens. *Yersinia pestis* is mainly transmitted by flea vectors, particularly *Xenopsylla* spp., but can also be transmitted mechanically by other flea species and hematophagous arthropods such as ticks and lice during epizootic periods [1, 2]. The bacteria infect mammals and rodents, which are considered to be the natural reservoirs once they survive the initial infection. The bacterium is believed to have originated from Central Asia, Transbajkalian and Mongolian steppes in the former Soviet Union (FSU) and spread out globally [3, 4]. *Yersinia pestis* has caused one of the most devastating historical pandemics of the world. It has caused three plague pandemics that affected many people, who succumbed to the infection [5]. Though these pandemics originated from the same place, Justinian plague strain was constrained to be the direct ancestor of those associated with the second and third pandemics, also known as the Black Death and the Oriental plague, respectively. The Black Death pandemic was the result of a separate emergence of *Y. pestis* from rodents into the human population and it gave birth to subsequent *Y. pestis* infections, including those that were responsible for the Oriental plague. The Black death spread in Europe and Africa, and probably back to China and the causal agent re-emerged as a new strain causing the outbreaks of plague that followed [6]. The third plague pandemic, probably started in the Yunnan province of China [7]. This episode spread to Hong Kong and later established fresh rodent foci in Asia, Africa, and North America, giving rise to an extant strain of the bacterium [8].

In Central and Southern Africa, there were possibly two routes in which the spread of the disease could have transpired. The first route of strain 1.ANT group in the second pandemic came through Egypt down to East and Central Africa, including the Uganda and Republic of Congo (Congo- Brazzaville). The second route of 1.ORI group could be in the third pandemic and spread through Madagascar and Southern Africa during the nineteenth century. It has been documented by Ziwa et al. [9], that plague was introduced to Eastern, Central and Southern Africa from the Middle East or the Far East by medieval traders, including slave and ivory caravans or via pilgrims to and from Egypt and Saudi Arabia.

**Fig. 1:**
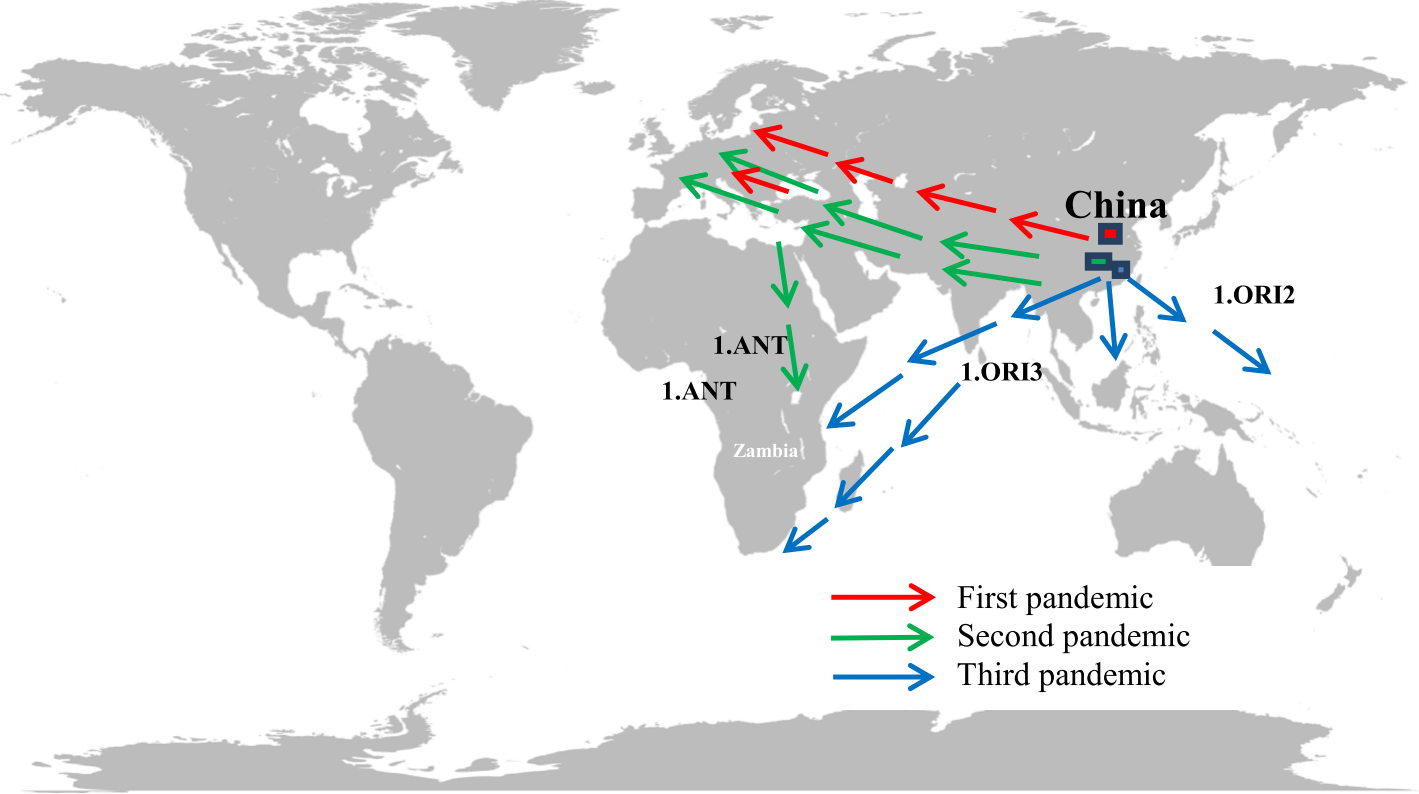
Geographical spread of *Yersinia pestis* from the suspected sources to Africa

Zambia, being a landlocked country, could probably be the intersection of the two routes of transmission, and it is most likely that the two different strains of the bacteria, 1.ANT and 1.ORI groups are present in this country (Fig. 1). Zambia experienced plague outbreak in three zones, namely: Eastern, Southern and North western [10–12]. In the Eastern part of the country, four districts have reported Plague outbreaks since 1917. These are Chama, Lundazi, Sinda and Nyimba [11]. However, in the past 15 years, the disease has occurred in the latter two districts, with the latest outbreak occurring in the Nyimba district in March 2015 [13]. No efforts have been made so far to identify the biotype or biovars of the bacterium in the area. The objective of this study was, therefore, to investigate the molecular epidemiology of plague among different potential reservoir hosts and their flea vectors in Sinda and Nyimba districts.

There are three biotypes (biovars) of *Y. pestis,* which can also be recognised by their distinct biochemical characteristics in their ability to reduce nitrates (NO_3_^**−**^) to nitrites (NO_2_^−^) and ferment glycerol. Biotype Antiqua is positive for both of the biochemical characteristics, Orientalis biovar forms nitrites from nitrates but does not ferment glycerol because of a 93 bp deletion in the glycerol 3-phosphate dehydrogenase *(glpD)* gene, while Medievali*s* biovar ferments glycerol, but does not form nitrites from nitrates due to a G to T nucleotide mutation that results in a stop codon in the *napA* gene [14, 15].

Based on the whole genome sequences to discover Single Nucleotide Polymorphisms (SNP), a global analysis demonstrated that the three *Y*. *pestis* strains could be separated into several populations with distinctive geographic patterns, including 0.ANT (Antiqua; Asia), 0.PE (Pestoides in group of Antiqua; Angola and Asia), 1.ORI (Orientalis; North and South America, Madagascar, Southeast Asia), 2.MED (Mediaevalis; Asia), 1.ANT (Antiqua; East and Central Africa), and 2.ANT (Antiqua; Asia), 3.ANT (Antiqua; China and Mongolia) and 4.ANT (Antiqua; Mongolia) [4, 6].

Strains of the three biotypes demonstrate no difference in their virulence or pathogenesis in animals and humans [16]. Regardless of the biotypes or biovars, the bacterium possesses three plasmids which contribute to its virulence namely; Calcium Dependant (pCD1), Murine Toxin/Fraction1 (MT1/pFra1) and Pesticin, Coagulase and Plasminogen activator (pPCP1). Plasmid PCP1 has 9.5 kb and is made up of five different genes namely: i) Pesticin (1074 bp *pst*), ii) Pesticin immunity- a transcriptional regulator gene (426 bp *pim*), iii) the replication regulation proteins (195 bp *rop*), iv) IS100 (made up of 1,02 kb transposase and 782 bp ATP-binding protein) and v) Plasminogen activator gene (939 bp *pla* gene) [17, 18]. The *pla* gene, only found in *Y. pestis*, is 1.4 kb and has 936 bp open reading frame (ORF). It is responsible for cleaving fibrin deposits that trap the microorganisms, consequently, helps to facilitate dissemination of the bacteria in the host. This gene is targeted for molecular diagnosis and epidemiology of the disease in this study as described elsewhere [19–21].

## Methods

### Study areas

This study was carried out in the Eastern part of Zambia in the two districts of Sinda and Nyimba, both of which have a recent history of the plague outbreaks. These areas experience high rainfall between the months of January and March [11]. The study was conducted between March 2015 and August 2016.

### Sample collection

#### Domestic animals

The villages were selected randomly and a maximum of 20 animals from each species, including pigs, goats and sheep, were sampled in each village upon obtaining the verbal consent from the livestock keepers (as per the approved Plague Research provided in Assurance No. FWA0000338). Depending on the species, each animal was assigned a unique number. The first number was picked randomly followed by a systematic technique (Systematic Random Sampling). Each restrained domestic animal was laid on a white plastic sheet and inspected for flea infestation. The animal was brushed with cotton wool soaked in 90% diethyl ether, to anaesthetise the ectoparasites, and then scrubbed with most an appropriate animal brush to remove fleas and other ectoparasites from its fur. Fleas fell onto the white plastic sheet and those which remained attached to the animal skin/fur were gently removed with a pair of fine forceps and put in small vials containing 70% ethanol. Animals below the age of six months and those which came from other villages, in the past six months, were excluded from the study.

#### Rodent and shrew trapping

Each selected village was divided into six arbitrary zones from which three zones were selected at random. Sherman’s live traps (50 × 65 × 157 mm) baited with peanut butter mixed with soya flour were set at a distance of 10 m apart in the nearby bushes and left overnight. Wire cage traps (145 × 100 × 230 mm) (Hoga-lab, Kyoto, Japan), baited with fish, *Stolothrissa tanganicae* (*Kapenta*)*,* and tomatoes, were set in selected houses in the zones. Traps were inspected the following morning and captured animals were taken to a nearby mobile laboratory for organ and flea collection. Trapping continued for three consecutive days in the same area as previously described elsewhere [13].

#### Collection of fleas from rodents and shrews

Fleas and other ectoparasites were collected from these animals by introducing the latter into the plastic bag with a cotton wool soaked in 90% diethyl ether. Once the animal and ectoparasites became anaethetise, they were transferred to a silver basin, where the animal was brushed with the toothbrush to disengage the fleas and other ectoparasites. Fleas which plummeted into the basin were gently picked using either a fine camel brush or a pair of forceps into serum vials with 70% ethanol, as a preservative. Other ectoparasites were also collected into separate vials containing 70% ethanol.

#### Collection of organs and bubo aspirates

Each rodent or shrew was aseptically dissected and organs (spleen, liver, lung, kidney and heart) were collected, divided into two parts in two separate vials and stored at −20 °C until required for use. After a verbal consent from the patients and/or their guardian, clinically suspected human plague cases were examined and about 0.5 ml of bubo aspirate was collected and inoculated into 5 ml of Brain Heart Infusion (BHI) medium (Oxoid, Hampshire, England).

### Flea identification

Fleas were pooled (1 to 5) according to their species and location, and from each pool, one to two fleas were removed, processed, and identified using main key features such as pronotal combs, genal combs, and the shape of head and reproductive organs (*spermathecae* in females and penis plates in males) as described by Kilonzo, [22].

### DNA extraction, PCR and sequencing

DNA extraction and PCR processing, from the rodent organs, human bubo aspirates and fleas, were performed as previously described [23, 24] and five positive samples were selected for the purpose of sequencing to determine their biovars or biotypes (Table 1).

**Table 1:** Flea vector species and their PCR results

District	Host/source of fleas	Host sampled (n)	Fleas collected	Species of fleas collected	SFI	No. of PCR positive
Nyimba	Rodents	120	15	*Xenopsylla cheopis*	0.12	3
	Shrews	17	0	–	–	–
	Pigs	2	3	*Ctenocephalides canis*	1.5	0
	Pigs	9	7	*Echidnophaga gallinacea*	0.8	4
	Goats	83	16	*Ctenocephalides canis*	0.19	5
Sinda	Pigs	121	382	*Echidnophaga gallinacea*	3.2	0
	Pigs	124	1064	*Echidnophaga larina*	8.6	0
	Goats	232	0	*–*	–	–
	Sheep	31	0	–	–	–
	Rodents	96	7	*Xenopsylla cheopis*	0.07	0
	Shrews	10	0	*–*	–	–
	Total	845	1494			12

DNA amplification was done using forward primers *Yp2 pla1* (5’ ATC TTA CTT TCC GTG AGA AG3’) and reverse primer Yp2 pla2 (5’ CTT GGA TGT TGA GCT TCC TA 3′), which amplifies 479 bp region corresponding to nucleotides 971 to 990 and 1431 to 1450 of the *pla* locus sequence, respectively [18, 25]. Using a Wizard_SV Gel and Clean-up system (Promega), PCR products were purifed following the manufacturer’s instructions. These products were sequenced with a BigDye Terminator Cycle Sequencing Ready Reaction Kit V3.1 and were further purified using ethanol/EDTA/sodium acetate precipitation, and separated on a 3130 Genetic Analyzer (Applied Biosystems). Assembling and editing of the obtained nucleotide sequences was accomplished by using GENETYX ATGC software, version 4.0.10 (GENETYX Co., Tokyo, Japan), and were equated with other *pla genes* of *Y. pestis* elsewhere using basic local alignment search tool (BLAST) in MEGA 6 software as described previously [26].

### Phylogenetic data analysis

Molecular evolutionary analyses were conducted using Molecular Evolutionary Genetics Analysis version (MEGA) version 6.0 [27]. Phylogenetic analysis of the derived sequences was performed with the *Y. pestis pla* gene reference sequences after an alignment was made via ClustalW. In the constructed phylogenetic tree, the sequences obtained from this study were compared with other reference sequences of *Y. pestis pla* gene acquired from the GenBank. This was accomplished by a homology search of *pla* gene of *Y. pestis* using BLAST technique [28]. The topological reliability of the trees was inferred by the bootstrap method with 1000 replicates.

## Results

### Molecular detection of *pla* gene of *Y. pestis*

A total of 216 rodents, 27 shrews, 245 pigs, 232 goats and 31 sheep were sampled for fleas, where a total of 22 fleas from rodents, 1456 fleas from pigs and 16 from goats, were collected. There were no fleas collected from shrews and sheep. Organs were collected from the rodents (*n* = 216) and the shrews (*n* = 27). Seven human bubo aspirates were also collected. Altogether, *Echidnophaga larina (*Jordan & Rothschild) (*n* = 1064), *Xenopsylla cheopis* (Rothschild) (*n* = 22), *Echidnophaga gallinacea* (Westwood) (*n* = 389) and *Ctenocephaides canis* (Curtis) (*n* = 19) were collected from the two districts.

The *pla* gene was detected in 6.9% rodents, 3.7% shrews, 42.9% human bubo aspirates and 0.94% fleas (Tables 1 and 2). The results also show that *Mastomys natalensis* had the highest number of positive *Y. pestis pla* gene (Table 3).

**Table 2:** PCR results from rodents, shrews and humans

District	Animal spp	No. of animals sampled	PCR positive	Positive (%)
Nyimba	Rodents	120	6	5.0
	Shrews	17	1	5.9
	Human	7	3	42.8
Sinda	Rodents	96	9	9.4
	Shrews	10	0	0
	Total	250	19

**Table 3:** PCR results of tissues from rodents (per specie) and shrews

Rodent spp	Sinda district		Nyimba district	
	No. sampled	No. positive (PCR)	No. sampled	No. positive (PCR)
*Mastomys natalensis*	52	5	68	5
*Gerbillurus* spp	22	3	0	0
*Rattus rattus*	19	0	34	1
*Crocidura* spp	10	0	19	1
*Saccostomus* spp. (Pouched mouse)	3	1	31	0
*Steatomys parvus* (Fat mouse)	0	0	5	0
Total	106	9 (9.4%)	137	7 (5.1%)

### Sequencing and phylogenetic analysis

**Fig. 2:**
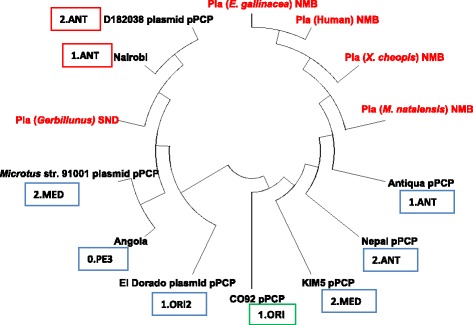
Dendrogram showing the evolutionary relationship of *Yersinia pestis* strains from Sinda and Nyimba districts using the Maximum Likelihood clustering method. ANT = Antiqua, MED = Medievalis, ORI=Orientalis, PE = Pestoide, NMB=Nyimba, SND = Sinda

From the 31 positive amplicons for *pla* gene of *Y. pestis*, five amplicons one from each species were selected for sequencing; *Homo sapiens* (Human), *E. gallinacea*, *X. cheopis*, *Mastomys natalensis* and *Gerbillinus* spp. (Table 4). A phylogenetic tree was constructed with the known strains of *Y. pestis pla* gene, which included: Antiqua CP009903.1 (1.ANT), Nairobi CP010294.1 (1.ANT), Nepal 516 CP000307.1 (2.ANT), CO92 L109969.1 (1.ORI), KIM F053945.1 (2.MED), Angola CP009936.1 (0.PE3), Eldorado CP009782.1 (1.ORI2), Microtus 9001 AE017046.1 (2.MED) and *Yersinia pestis* D182038 CP001592.1 (2.ANT) strains. (Nucleotide sequences of the *pla* gene of *Y. pestis* from different hosts from this study are found in Additional file 1:Table S1). Strains from Nyimba demonstrated a high degree of similarity with *Y. pestis* of Antiqua (1.ANT) (Accession No. CP009903.1), isolated from a human in the Republic of Congo. In contrast, the Sinda strain showed that it was (98%-99%) related to the Nairobi strain (Accession No. CP010294.1), isolated from wild rodent in Nairobi, Kenya (Fig. 2).

**Table 4:** Isolation frequency and host distribution of *Y. pestis* isolates in this study

Host		Species	No. of *Y. pestis* DNA extracts	District
Host	Rodent	*Mastomys natalensis*	5	Nyimba
		*Mastomys natalensis*	5	Sinda
		*Gerbillurus* spp	3	Sinda
		*Rattus rattus*	1	Nyimba
		*Crocidura* spp	1	Nyimba
		*Saccostomus* spp	1	Sinda
	Human	*Homo sapiens*	3	Nyimba
Vector	Fleas from Goat	*Ctenocephalides canis*	5	Nyimba
	Fleas from Pigs	*Echidnophaga gallinacea*	4	Nyimba
	Fleas from Rodents	*Xenopsylla cheopis*	3	Nyimba
		Total	31

## Discussion

The presence of *pla* gene of *Y. pestis* in rodents, fleas, a shrew and human samples was an indication that the bacterium was present and was the cause of the disease outbreak in Nyimba, where sampling was done just after an outbreak of plague. The results showed that during the outbreak different species of flea vectors have the potential to acquire and mechanically transmit the bacterium to other animals as previously described [29]. In contrast, in Sinda district, the flea vectors were negative for *pla* gene, suggesting that during the quiescent period, fleas did not harbour the bacterium, although the latter was still isolated from the rodents, which are the natural reservoirs of *Y. pestis*. It is, therefore, established that the bacterium was in circulation among the reservoir hosts and the disease could break out anytime if environmental conditions for both the hosts and flea vectors, become favourable.

From our findings, it is demonstrated the isolates from Nyimba districts were closely related to those of Antiqua (1.ANT) from the Republic of Congo, isolated from human samples. The Nyimba isolates may have originated from China and migrated during the trade voyages as the steamships sailed from the infected regions to non-infected areas of the world [30]. This is consistent with the findings by Morelli et al. [4], that the isolates in the East and Central Africa involve the 1.ANT group, which they estimated was between 628 and 6914 years and predated the trade voyages in China [8, 31]. Intercontinental trade carried large consignments of cereals which provided a suitable environment for rodents harbouring flea-vector, *Rattus rattus* and *Xenopsylla cheopis* respectively, which are primary natural reservoirs and vectors of the *Y. pestis* [30].

The Sinda isolate was closely related to the Nairobi strain [32] (Fig. 2). This entails that the strain may have originally spread from China to East Africa through the Nile route of Arab traders down south to Central Africa during the second pandemic [4, 26]. Both strains may have migrated forth south probably along the Great Rift Valley during the seasonal migration of rodents along Rukwa valley down to Luangwa valley [33]. The plague outbreak in Nyimba occurred in the Luangwa valley, whereas Sinda is a few kilometres from the Valley but closer to Mozambique. As previously described by Davis, [34], there was a similarity between the Luangwa Valley rodent species and those in Southern Tanganyika (current Tanzania) and Nyasaland (current Malawi) near the border with Northern Rhodesia (current Zambia). Our findings are consistent with those described by Davis et al. [33], that the strain isolated from Mukomba in Lundazi district in eastern Zambia, were biochemically glycerol positive and reduced nitrates to nitrites indicating that it was Antiqua strain of *Y. pestis* isolated [15]. This bacterium may have been transported along the valley from Northern to Southern Luangwa by either rodents or by human migration [30, 35].

## Conclusions

It is concluded that *Y. pestis* was present in the study area and the isolates from Nyimba district were similar despite coming from different hosts or sources. The Nyimba isolates demonstrated a high degree of evolutionary relationship with Antiqua (1.ANT) strain from the Republic of Congo, while the Sinda strain was evolutionary similar to Nairobi (1.ANT) strain in Kenya.

## Additional file

Additional file 1: Table S1. Nucleotide sequences of *pla* gene of *Y. pestis* from this study. (DOCX 34 kb)

## Abbreviations

BLAST: Basic Local Alignment Search Tool
MEGA: Molecular Evolutionary Genetic Analysis
PCR: Polymerase Chain Reaction
