# Supplementary material for: Molecular epidemiological investigations of plague in Eastern Province of Zambia
Source: BMC Microbiol. 2018 Jan 4;18:2. doi: 10.1186/s12866-017-1146-8 (PMC5810007; doi:10.1186/s12866-017-1146-8)
Supplement: Supplementary file 1 — Nucleotide sequences of pla gene of Y. pestis from this study. (DOCX 34 kb) [file 12866_2017_1146_MOESM1_ESM.docx]

Table S1: Nucleotide sequences of *pla* gene of *Y. pestis* from different hosts (**Additional file 1**)

| **Name of district** | **Host** | ***Y. pestis* gene targeted** | **Nucleotide** | **Country** | **Accession number** | **Reference** |
| --- | --- | --- | --- | --- | --- | --- |
| Sinda | Rodent- *Gerbillurus* spp | Pla | 5'TAATTTGACCTTCTCCATGCCCTGAAAGACGTGGAGAATGTCAAGGCAAAACAACAAAATGAGCGCCCCGTCATTATGGTGAAAAAGGTGATTTTTCACACATCTGAAGGTTGGTCAAACACCAACCTCCTTTCCACAGACATCCTCCCCGCTAGGGGAGGATGAAAAGAGAGATATGATCTGTATTTTTCAGAAGCGATATTGCAGACCCGCCGTCACAGTATAATTTTTATTGGAAATACCGGCAGCATCTCCGCCAATAGAGACAGAATCTCCACTATTCTTATCAATGGTCTGAGTACCTCCTTTGCCCTCATCATATTTACTGTATGTAAATTCCGCAAAGACTTTGGCATTAGGTGTGACATAATATCCAGCGTTAATTACGGTACCATAATAACGTGAGCCGGATGTCTTCTCACGGAAAGTAAGA3' | Zambia | This study | This study |
| Nyimba | Rodent –*Mastomys natalensis* | Pla | 5′CCGTGAGAAGACATCCGGCTCACGTTATTATGGTACCGTAATTAACGCTGGATATTATGTCACACCTAATGCCAAAGTCTTTGCGGAATTTACATACAGTAAATATGATGAGGGCAAAGGAGGTACTCAGACCATTGATAAGAATAGTGGAGATTCTGTCTCTATTGGCGGAGATGCTGCCGGTATTTCCAATAAAAATTATACTGTGACGGCGGGTCTGCAATATCGCTTCTGAAAAATACAGATCATATCTCTCTTTTCATCCTCCCCTAGCGGGGAGGATGTCTGTGGAAAGGAGGTTGGTGTTTGACCAACCTTCAGATGTGTGAAAAATCACCTTTTTCACCATAATGACGGGGCGCTCATTCTGTTGTTTTGCCTTGACATTCTCCACGTCTTTCAGGGCATGGAGAAGGTCAAATTAGACATGGAACGCTACTCTCCTTCCTGTAGGAAGC3′ | Zambia | This study | This study |
| Nyimba | Flea- *Xenopsylla cheopis* | Pla | 5′CCGTGAGAAGACATCCGGCTCACGTTATTATGGTACCGTAATTAACGCTGGATATTATGTCACACCTAATGCCAAAGTCTTTGCGGAATTTACATACAGTAAATATGATGAGGGCAAAGGAGGTACTCAGACCATTGATAAGAATAGTGGAGATTCTGTCTCTATTGGCGGAGATGCTGCCGGTATTTCCAATAAAAATTATACTGTGACGGCGGGTCTGCAATATCGCTTCTGAAAAATACAGATCATATCTCTCTTTTCATCCTCCCCTAGCGGGGAGGATGTCTGTGGAAAGGAGGTTGGTGTTTGACCAACCTTCAGATGTGTGAAAAATCACCTTTTTCACCATAATGACGGGGCGCTCATTCTGTTGTTTTGCCTTGACATTCTCCACGTCTTTCAGGGCATGGAGAAGGTCAAATTAGACATGGAACGCTACTCTCCTTCCTGTAGGAAGC3′ | Zambia | This study | This study |
| Nyimba | Flea- *E. gallinacea* | Pla | 5′CCGTGAGAAGACATCCGGCTCACGTTATTATGGTACCGTAATTAACGCTGGATATTATGTCACACCTAATGCCAAAGTCTTTGCGGAATTTACATACAGTAAATATGATGAGGGCAAAGGAGGTACTCAGACCATTGATAAGAATAGTGGAGATTCTGTCTCTATTGGCGGAGATGCTGCCGGTATTTCCAATAAAAATTATACTGTGACGGCGGGTCTGCAATATCGCTTCTGAAAAATACAGATCATATCTCTCTTTTCATCCTCCCCTAGCGGGGAGGATGTCTGTGGAAAGGAGGTTGGTGTTTGACCAACCTTCAGATGTGTGAAAAATCACCTTTTTCACCATAATGACGGGGCGCTCATTCTGTTGTTTTGCCTTGACATTCTCCACGTCTTTCAGGGCATGGAGAAGGTCAAATTAGACATGGAACGCTACTCTCCTTCCTGTAGGAAGC3′ | Zambia | This study | This study |
| Nyimba | Human *(Homo sapiens)* | Pla | 5′CCGTGAGAAGACATCCGGCTCACGTTATTATGGTACCGTAATTAACGCTGGATATTATGTCACACCTAATGCCAAAGTCTTTGCGGAATTTACATACAGTAAATATGATGAGGGCAAAGGAGGTACTCAGACCATTGATAAGAATAGTGGAGATTCTGTCTCTATTGGCGGAGATGCTGCCGGTATTTCCAATAAAAATTATACTGTGACGGCGGGTCTGCAATATCGCTTCTGAAAAATACAGATCATATCTCTCTTTTCATCCTCCCCTAGCGGGGAGGATGTCTGTGGAAAGGAGGTTGGTGTTTGACCAACCTTCAGATGTGTGAAAAATCACCTTTTTCACCATAATGACGGGGCGCTCATTCTGTTGTTTTGCCTTGACATTCTCCACGTCTTTCAGGGCATGGAGAAGGTCAAATTAGACATGGAACGCTACTCTCCTTCCTGTAGGAAGC3 | Zambia | This study | This study |
